# Rare Generalized Form of Fungal Dermatitis in a Horse: Case Report

**DOI:** 10.3390/ani10050871

**Published:** 2020-05-17

**Authors:** Barbara Padalino, Jeanine Rhoda Sandy, Roberta Barrasso, Adriana Trotta, Giancarlo Bozzo, Claudia Cafarchia

**Affiliations:** 1 Department of Agricultural and Food Sciences, University of Bologna, Viale Giuseppe Fanin 46, 40127 Bologna, Italy; barbara.padalino@unibo.it; 2 Veterinary Diagnostic Laboratory, City University of Hong Kong, 83 Tat Chee Avenue, Kowloon, Hong Kong; j.sandy@cityu.edu.hk; 3 Department of Veterinary Medicine, University of Bari, Strada Provinciale per Casamassima km 3, 70010 Valenzano (BA), Italy; adriana.trotta@uniba.it (A.T.); giancarlo.bozzo@uniba.it (G.B.); claudia.cafarchia@uniba.it (C.C.)

**Keywords:** fungal skin disease, health, equine, diagnosis

## Abstract

**Simple Summary:**

The study describes a rare case of generalized *Geotrichum SPP* dermatitis in a horse. A saddle horse showed well-circumscribed areas of non-pruritic alopecia and the owner, suspecting allergic skin disease, commenced a three-week course of corticosteroids. Clinical signs progressed and a veterinarian was called. At veterinary examination, the horse was lethargic and pyretic with easily epilated hairs and hind limb swelling. Diagnostic procedures included collection and analyses of blood, hair and skin cytology, and biopsy. *Geotrichum candidum* was isolated and the horse was successfully treated with an antifungal solution and antioxidants. This study may be useful for owners and veterinarians because it describes in detail clinical signs, diagnostic procedures, and treatment of a rare case of generalized *Geotrichum* infection.

**Abstract:**

A rare case of *Geotrichum* spp. dermatitis in a horse is presented. After unrelated, previous surgery and antibiotic treatment, a saddle horse showed well-circumscribed areas of non-pruritic alopecia. Suspecting allergic skin disease, the horse was treated with corticosteroids. The skin lesion spread, and a second veterinarian was consulted. At clinical examination, the horse was lethargic, pyretic and hair was shedding/easily epilated over the head, neck, shoulders, and legs and the hind legs were swollen. Blood analysis revealed mild leucocytosis and hyperglobulinemia. Hair, skin scraping, and skin biopsy ruled out parasites and bacteria; cytology identified yeast-like structures with hyphae or pseudohyphae. *Geotrichum candidum* was isolated on culture. Treatment consisted of stable disinfection, topical application of an antifungal solution, vitamins C and E supplementation and allowing the horse to graze in sunlight for at least 6 h/day. At 3-weeks follow-up, the horse had gained weight, alopecia was decreased, and all other clinical parameters were normal. Antifungal treatment was continued twice a week for three months. This study suggests *Geotrichum candidum* may cause skin lesions in horses after long-term use of corticosteroids or antibiotics. To avoid unnecessary and prolonged suffering in cases of dermatitis, veterinarians should be promptly consulted, appropriate diagnostic procedures conducted, so that a definitive diagnosis can be reached, and an appropriate treatment regimen implemented.

## 1. Introduction

Skin disease can adversely affect the health and welfare of horses, and disease can be exacerbated by delays in diagnosis and commencement of suitable treatment regimens, also not helped by clinical signs often being similar despite causes being different (infectious versus non-infectious) [1]. Infectious skin disease can be caused by bacteria, parasites, and fungi [2]. Skin diseases caused by fungi are called mycoses and can be categorized as dermatophytosis and dermatomycosis. Dermatophytosis involves infection of the keratinized tissues including hair and stratum corneum, caused by fungi such as *Microsporum*, *Trichophyton,* or *Epidermophyton*. Dermatomycoses are fungal infections of hair, claw, or skin caused by non-dermatophyte fungi, not classified in the aforementioned genera [3]. Fungal skin infections can also result from widely disseminated internal infections. Fungal diagnosis requires a thorough description of the clinical signs, as well as accurate identification of the fungal organism; both are crucial for positive treatment outcomes. Equine fungal diseases can also be classified into superficial, cutaneous, subcutaneous, and deep mycoses [4]. Cutaneous infections are the most common fungal skin infections in horses, with dermatophytoses and onychomycoses commonly reported [5]. Dermatophytoses are superficial, cutaneous mycoses caused by dermatophytes and these diseases are considered as zoonoses. *Geotrichum candidum* is an etiological agent of equine dermatomycosis. In a study by Figueredo et al. (2011) [6], 28.1% of the horses with a suspected fungal infection, was due to *Geotrichum candidum*. Those horses showed dry, erythematous, well defined circular alopecia in 92% of the cases and more rarely, desquamation and pruritus. Onychomycosis is a fungal infection of the hoof horn, commonly secondary to deterioration and disruption of the horn wall integrity [7] often due to *Scedosporium* spp., *Trichophyton* spp. and *Scopulariopsis brevicaulis* [8,9]. Other dermatomycoses are uncommonly diagnosed in horses. From the authors’ knowledge, detailed descriptions of generalized *G. candidum* dermatitis has not been previously reported.

Fungal infections are potentially zoonotic and represent challenging cases to accurately diagnose. Other than superficial dermatophytosis (ringworm), for which culture and direct microscopic examination of hair shafts and skin scrapings generally lead to a fast, accurate diagnosis, all other fungal infections, excluding the use of cytology, often require invasive, expensive and slow diagnostic techniques such as biopsy and culture [5]. As a result, fungal infections can be neglected by public health authorities and by the greater scientific community, resulting in scant availability of epidemiological data and poor general knowledge of these infections.

The current study documents a rare case of *Geotrichum candidum* dermatomycosis in a horse, which caused chronic skin disease and significant morbidity, partly attributed to incorrect clinical management. The goal of this case report is to educate horse owners, trainers and veterinarians to consider utilizing various diagnostic tools to enable accurate diagnosis of disease, especially where the initial clinical response to treatment is poor.

## 2. Materials and Methods

### 2.1. History

**Figure 1:**
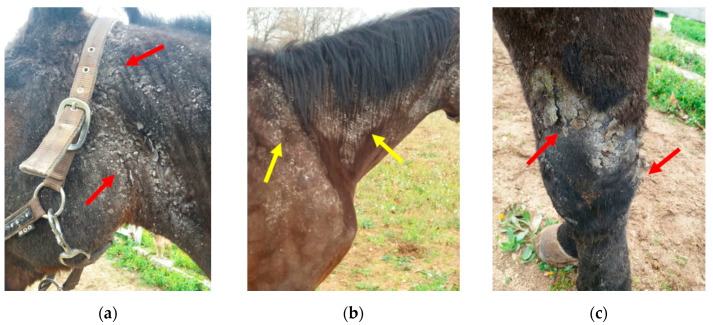
Distribution of alopecia and crusting seen during the second veterinary examination. (**a**) Alopecic areas, with thick keratin-rich crusts, distributed over the head; (**b**) alopecia of the neck (with sparing over the trachea/ventrum) and shoulders; (**c**) skin erosion on the left knee (palmar and lateral).

At the beginning of the winter season, an eleven-year-old gelded bay saddle horse exhibited well-circumscribed, non-pruritic nodules, 1–2 cm in diameter, on the dorsal part of the neck. Those lesions appeared one week after standing surgery to remove a fracture from the lateral splint bone for which the patient was placed on prophylactic post-surgical antibiotic treatment (gentamicin 6 mg/kg BW; and procaine penicillin 8.000 U.I./kg BW for 7 days). The horse was housed night and day in a single box (3.5 × 3.5 m), with a small window, in an indoor horse stable hosting about 20 horses. The horse wore a winter waterproof wool blanket with neck cover to stay warm and was bedded on straw. On development of these skin lesions, the owner did not consult with a veterinarian, as the lesions were suspected to represent an allergic reaction, so the owner administered corticosteroids (dexamethasone) per os for a week. The owner monitored the horse during treatment and no skin improvement was noted, and the horse lost weight (about 50 Kg BW and one point in Body Condition Score (BCS) over 3 weeks, the latter attributed to reduced appetite. One week after cessation of oral steroids, a veterinarian was consulted. The veterinarian reported alopecic skin nodules and well-circumscribed, non-pruritic alopecia on the upper lateral region of the front legs. Parental corticosteroids (prednisolone, 1 mg/kg BW) and antihistamine (chlorphenamine, 5 mg/kg BW) were prescribed for a week, as an allergic reaction was still clinically suspected. During this second treatment regimen and continuing for an additional two weeks, dermatitis spread to the head, shoulders and legs (Figure 1), the horse remained inappetant and became lethargic, with clinical signs of colic (i.e., pawing, stamping) which were then treated with flunixin meglumine (1.1 mg/kg BW iv). At this time, a second veterinarian was consulted, approximately one month after the initial nodules developed over the neck.

### 2.2. Clinical Examination and Sampling

During the physical examination performed by the second veterinarian (B.P.), the horse was lethargic, pyretic (40 °C), with increased heart and respiratory rates (54 bpm and 28 bpm, respectively) and hair was shedding/easily epilated over the head, neck, shoulder, chest and all four legs, with severe swelling of the hind limbs and carpal region. Yellow exudate could be easily expressed from the surface of a skin erosion (2 cm) on the left knee (palmar and lateral). The distribution of alopecia involved the head (severe on the cheek and forelock regions, with sparing over the nose, nostril and muzzle regions), neck (widespread with sparing over the trachea/ventrum) and circumferentially around all four lower limbs and shoulders, most severe between the neck and shoulder regions. Large white, loosely adherent scale coated alopecic areas, with thick keratin-rich crusts, ranging from 0.1 to 1 cm (Figure 1).

Blood was collected via the external jugular vein into two tubes with ethylenediaminotetracetic-acid (EDTA) and without anticoagulant of the vacutainer system (Becton Dickinson, Franklin Lakes, NJ, USA), stored at 4 °C and sent to the Laboratory of Internal Medicine (University of Bari, Italy). Hair and skin scraping samples were collected from lesions (at the neck level) using a sterile scalpel and pliers and placed into plastic vials containing 70% ethanol and submitted for parasitological analysis to the Laboratory of Parasitology of the Department of Veterinary Medicine (DVM) (University of Bari, Italy). Hair was collected by brush and also by tweezers making sure to collect hairs with bulbs. Hair and skin scraping samples were placed into a sterile petri dish and sent to the Mycology laboratory of DVM. Anti-inflammatory drugs were administered (flunixin meglumine 1.1 mg/Kg iv, once daily for three days). A week after this initial physical examination, additional samples were collected. Three skin biopsies (1 cm × 1 cm each) were performed from three different sites over the neck and included a margin of a recently developed lesion. Biopsy samples were placed into a sterile tube and sent at 4 °C to the Laboratories of Pathology and Bacteriology, DVM for histopathology and culture. The same day, blood samples and hairs were collected and sent for bacteriological and mycological analysis to the Laboratories of Bacteriology and Mycology of DVM.

#### 2.2.1. Hematology

Blood cell counts (BCC) were performed using the Abbott cell counter analyzer CELL DYN 3700 (Abbott, Chicago, IL, USA) within 4 h of sampling and remaining blood was stored at 4 °C. Serum was obtained by centrifugation (1600× *g* for 15 min) and biochemical parameters were assessed with ASSEL reagents and the SEAC photometer with interferential filters. Serum protein electrophoresis was performed on an agarose gel, according to the Helena BioSciences method [10].

#### 2.2.2. Parasitological, Bacteriological and Mycological Analysis

Parasitological analysis of the skin scraping utilized a stereomicroscope within 48 h of sample collection. For bacteriological analysis, skin biopsies and five microliters of blood were plated onto Columbia blood agar, McConkey agar, Mannitol salt agar and Tryptic Soy Broth (Liofilchem, Teramo, Italy) and incubated at 37 °C for 48 h under anaerobic, aerobic and microaerophilic conditions. For mycological investigation, five microliters of blood were cultured onto Bact/Alert PF PLUs and incubated at 37 °C for 7–14 days. Mycological analysis utilized hair and skin scraping samples stained with May-Grünwald Giemsa (M.G.) and fresh samples were cultured onto Sabouraud dextrose agar plates with chloramphenicol (0.4 mg/mL) and cycloheximide (0.5 mg/mL), incubated at 25 °C for 7–14 days. Fungi were isolated and identified based on macroscopic and microscopic features [11]. Api 20C AUX test (bioMerieux, Marcy L’etoile, France) were used according to the manufacturer’s instructions to confirm the morphological identification of the fungus.

#### 2.2.3. Cytology and Histopathology

Impression smears made from fresh skin biopsy material and hair samples were examined cytologically after fixation in methanol for 15 min and stained with M.G. Skin biopsy material was fixed in 10% neutral buffered formalin and routinely processed into paraffin wax and 4 µm sections were stained with Hematoxylin and Eosin (H.E.), Periodic Acid-Schiff (PAS) and Grocott Methenamine Silver (GMS) [12]. The slides were sent to the Veterinary Diagnostic Laboratory, City University of Hong Kong, for consultation.

## 3. Results

### 3.1. Hematology/Biochemistry

**Figure 2:**
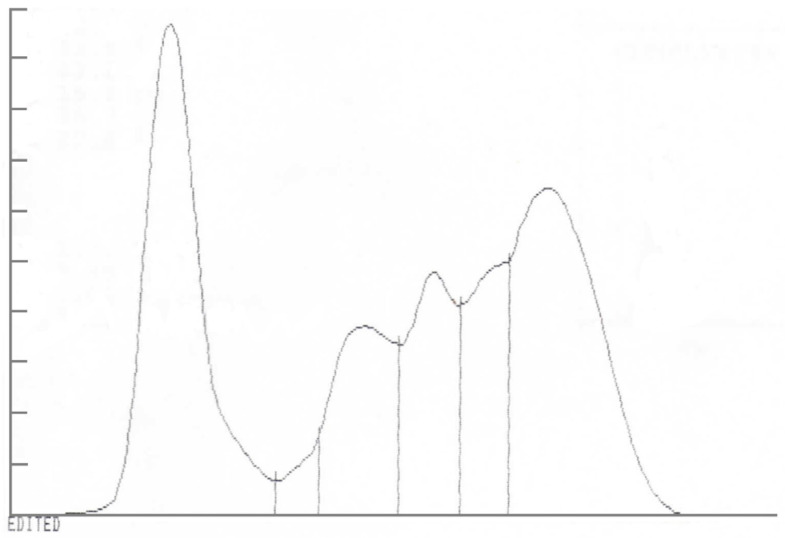
Hypergammaglobulinemia on serum protein electrophoresis from a horse affected by dermal geotrichosis for two months.

The BCC parameters were within the normal range, apart from a minimal increase in leucocytes due to neutrophilia (Table 1). LDH, bilirubin, urea, and triglycerides levels were mildly increased (Table 2). Total proteins were mildly elevated, due to a mild increase in globulins representing hypergammaglobulinemia as per electrophoresis. Alpha 1 fraction was moderately decreased and both beta fractions were mildly to moderately increased (Figure 2, Table 3).

### 3.2. Parasitology, Bacteriology, and Mycology

**Figure 3:**
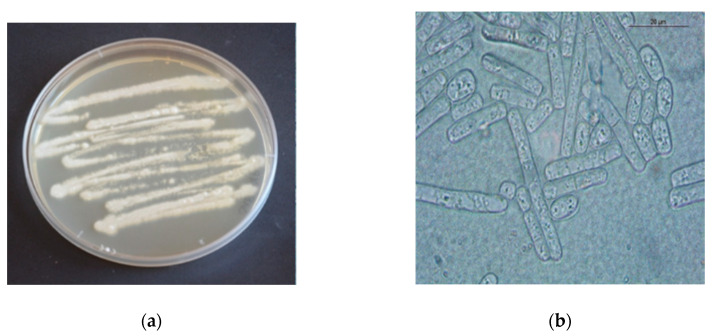
Macroscopic (**a**) and microscopic (**b**) features of *Geotrichum candidum*. (**a**): White, dry, powdery to cottony colonies of *Geotrichum candidum*. (**b**): Hyaline septate hyphae breaking up into chains of hyaline, smooth, one-celled, sub-globose to cylindrical arthroconidia (6–12 × 3–6 µm in size).

Skin biopsies revealed normal contaminant microflora and the blood culture sample tested negative for bacterial growth after 48 h of incubation and negative for fungi after 14 days. Parasitological analyses were negative. *Geotrichum candidum* was cultured from skin scale (Figure 3). The fungal culture was repeated twice from hair samples collected on two different days and *Geotrichum candidum* was the only fungus isolated in pure culture.

### 3.3. Cytology

**Figure 4:**
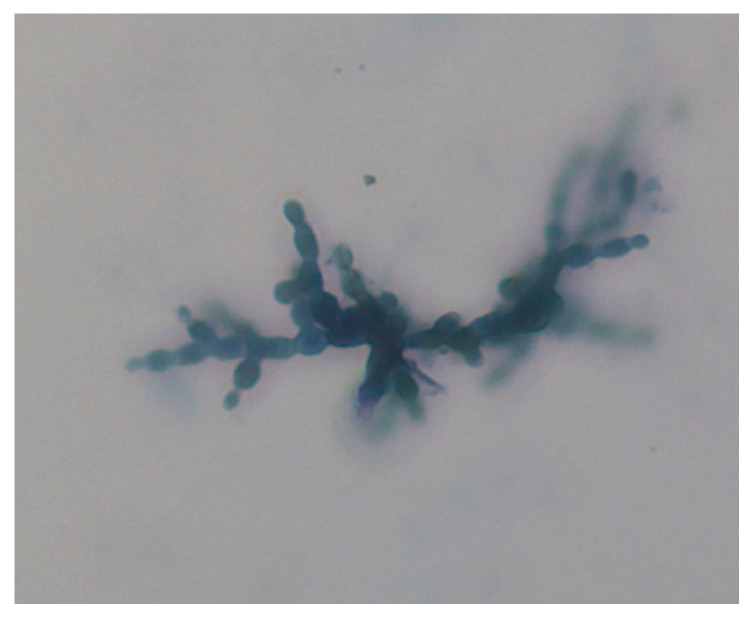
Cytology impression smear from hair and skin sample collected from a horse with severe dermatitis; May-Grünwald Giemsa stained specimen revealing a mass of hyphae (M.G. 100×, oil objective).

Cytology revealed a mass of branching segmented hyphae forming rectangular or oval-shaped arthroconidia which stained blue (M.G.) and varied in length from 3 to 6 μm (Figure 4).

### 3.4. Histopathology

**Figure 5:**
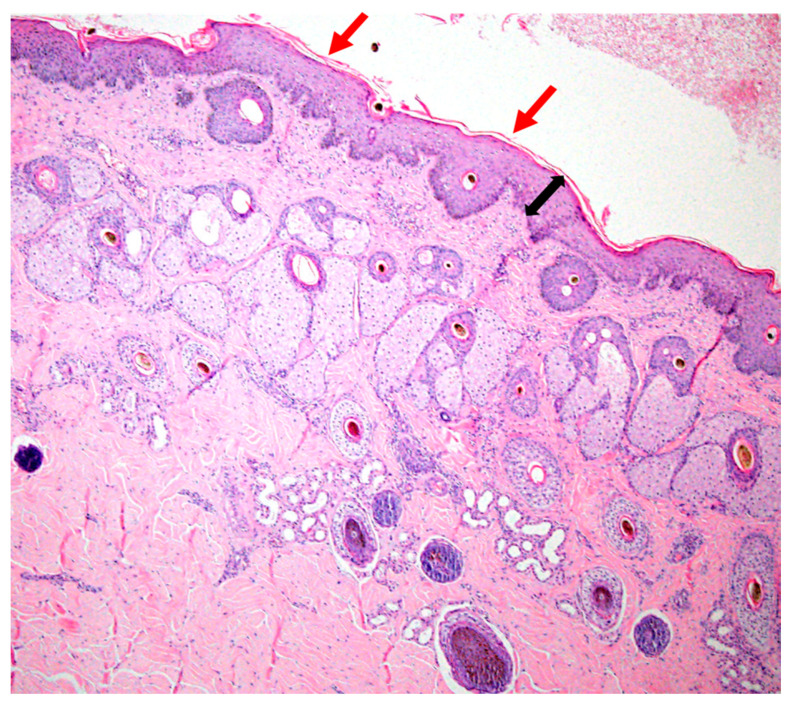
Skin histopathology from a horse with severe dermatitis; mild lymphocytic, hyperplastic dermatitis (H.E. 4× objective). Black arrow indicates hyperplasia (acanthosis) of the epidermis. Red arrows indicate mildly increased surface keratin.

**Figure 6:**
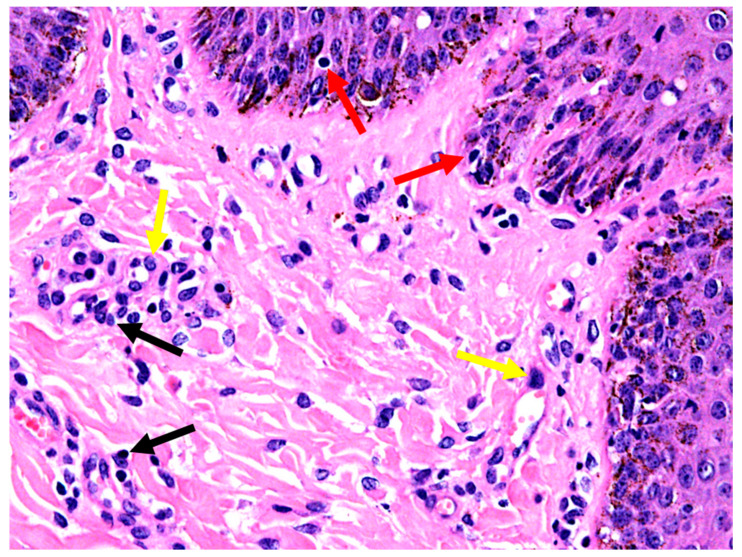
Skin histopathology from a horse with severe dermatitis, showing mild lymphocytic, hyperplastic dermatitis (H.E. 40× objective). Black arrows highlight perivascular inflammatory cells; yellow arrows highlight plump endothelial cells of dermal capillaries; red arrows highlight leucocytes in epithelium.

A single biopsy sample of haired skin exhibited mild orthokeratotic hyperkeratosis with mild epidermal hyperplasia due to acanthosis. There was a minimal degree of interface dermatitis, with low numbers of leucocytes, which were difficult to further identify, crossing the epidermis. The superficial dermis exhibited a mild perivascular inflammatory pattern with small lymphocytes admixed with fewer neutrophils. Endothelium was plump and reactive and small caliber dermal capillaries were moderately dilated. Hair follicles were in different stages of the hair cycle with retained hairs and anagen bulbs seen (Figure 5 and Figure 6). PAS and GMS did not reveal any fungal organisms. No surface crust material was submitted.

### 3.5. Histopathological Diagnosis

Mild perivascular to interface lymphocytic, hyperplastic dermatitis (Figure 5 and Figure 6).

### 3.6. Treatment

Therapy using a topical solution (2% of concentration) of miconazole and chlorhexidine [15], applied once a day for a week, then every other day for 14 days, and finally twice a week for 28 days, was recommended [5]. The patient’s diet was supplemented with vitamins C (ascorbic acid, 30 g/daily *per os*) and E (5000 IU/daily *per os*). The indoor stable and horse equipment were disinfected (Virkon S Farmaceutici Gellini, Aprilia, Italy) [6] and the management was changed, providing at least 6 h/day of grazing without a rug.

### 3.7. Follow Up

**Figure 7:**
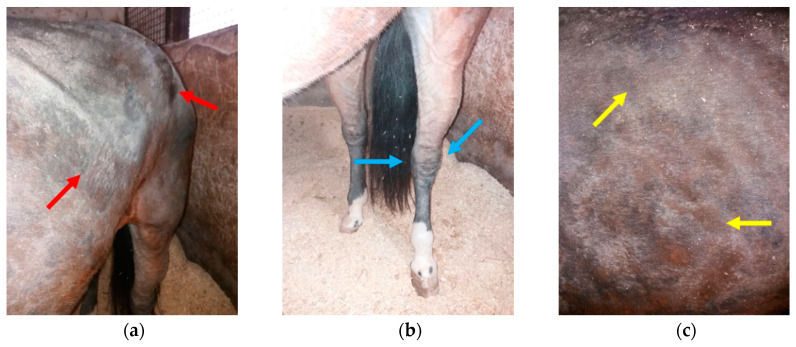
Gross photos of the horse at three-week follow-up. Alopecic regions showed new hair growth with fewer crusts on the abdomen and back (**a**), tarsal area (**b**) and thorax (**c**).

Three-weeks following topical treatment, the horse had gained weight (about 80 Kg difference using a BW tape) and BCS (manually evaluated), alopecic regions showed new hair growth with fewer crusts (Figure 7), hematological and biochemical parameters returned to the normal range [13,14]. The antifungal treatment was continued twice a week for three more months. The horse recovered completely, and antioxidant supplementation was recommended as an ongoing dietary supplementation as prescribed.

## 4. Discussion

This case report documents a case of dermal geotrichiosis in a horse previously treated with prophylactic, post-surgical antibiotic and corticosteroid therapy. The horse was housed in a single box for 24 h wearing a winter blanket and then improperly treated with corticosteroids for three weeks (by the owner for a week and then by the first of two veterinarians, for two weeks). Corticosteroid treatment may have impaired the immune system of this horse, making the horse suspectable to contracting fungal infection. Fungal dermatitis secondary to immunosuppressive dose rates of corticosteroids has been previously reported in dogs [16] and red flamingos [17]. This case report highlights the importance of collecting appropriate samples to enable a thorough clinical workup of skin diseases in horses. Due to the initial lack of appropriate treatment when the skin disease first developed, this horse experienced prolonged sufferance for more than two months, developing chronic, spreading skin disease, with systemic signs of illness including weight loss, inappetence, fever, and colic. Equine skin diseases are often challenging to diagnose and successfully treat, but this case demonstrates that appropriate laboratory tests should always be conducted as part of the basic principles of responsible pet ownership and proper veterinary duty of care, which are important to safeguard animal health and well-being.

The housing conditions of this horse may have been beneficial for fungal infection to become established. The waterproof wool blanket may have created a humid microenvironment and increased sweat production as previously reported [18], favoring the growth and spread of the fungus. Moreover, the horse was stabled day and night, with little exposure to direct sunlight, which is vital for vitamin D production, important to keep skin healthy [19]. Glucocorticoid and antibiotic usage, and perhaps some debilitation induced from the previous surgery, may have further predisposed the horse to developing fungal infection. The failure to identify the disease early in the disease course, also played a role in the development of extensive skin disease in this case, as dermatomycoses, are generally considered to represent opportunistic disease [5]. It was difficult to decide whether all the clinical signs revealed at the second clinical examination were due to the dermal geotrichosis, or whether a systemic disease process was complicating the clinical picture. Only skin bacteriological and parasitological tests were run, and those possible skin diseases were excluded because negative. However, since specific tests to detect systemic infections were not performed, it would be impossible to be certain whether dermal geotrichosis was the primary condition or represented an opportunistic infection with a horse with co-morbidity.

The biochemical profile supported chronicity of inflammation due to the elevated globulins, low albumin/globulin ratio (useful as a biomarker of inflammation), with inflammation further supported by neutrophilia [14]. Serum electrophoresis was consistent for a polyclonal gammopathy, likely due to increased IgG and IgM. The increased β1 fraction was assumed to be due to increased IgM and isotypes of IgG which can migrate into this region. Increased β2 fraction was likely due to isotypes of IgG, such as IgGT, and/or IgM, which can both spill over into this fraction. Confirmation via immunoelectrophoresis was not available to confirm this. The decreased α1 fraction is unexpected as this fraction is represented mostly by positive acute phase proteins. The decrease may indicate the chronicity of the case, confirming the late diagnosis. This decrease may also indicate protein loss from skin exudation and/or loss from the gut, which may also have contributed to the low albumin value, although albumin is typically reduced in inflammatory states [14]. The increase in LDH, urea, and triglycerides and low magnesium mirror the negative energy status of the horse, which was refusing to eat and showed mild signs of colic the day before blood sampling [13].

*Geotrichum* spp. are yeast-like fungi, commonly isolated from soil, air, water, milk, silage, and plant tissues and can be isolated from the digestive tract, skin, vagina, and mouth of humans and other mammals [20,21]. In some cases, this fungus can be pathogenic, producing disseminated or localized skin disease in both humans and animals [22,23,24]. There are 13 species of *Geotrichum* but *Geotrichum candidum* is one of the most important species reported to cause infection in animals [20,21]. *Geotrichum candidum* was isolated in 28.1% of the horses in a retrospective study of 64 horses presenting with skin lesions referable to fungal infection [6]. That study identified that the age of affected horses was a risk factor, with horses aged between 3 and 17 years more likely to become infected and develop lesions. The horse in the current study was 11 years old, so was within the age range described in the retrospective study. Figueredo et al. [6] reported that the head and neck regions were the most common regions affected with fungal dermatitis, whilst legs and tails were less likely affected. The current horse had severe alopecia particularly affecting the head and neck regions, which likely represented primary areas of infection, in agreement with the literature [6] and then, the fungus likely migrated or was spread to other parts of the body. In the retrospective study by Figueredo et al. [6] only 1/12 horses had generalized skin geotrichosis, similar to the current case, but details of the case were not described. This study is therefore useful to educate veterinarians on how to diagnose generalized *Geotrichum candidum* infection in horses.

Geotrichosis causes alopecia with desquamation and more rarely erythema, hyperkeratosis, and pruritus in horses [6]. The clinical signs in the current horse at the beginning were in agreement with the literature, with the horse becoming inappetant and pyretic. Likely, the extent of dermatitis and the lack of prompt treatment (i.e., chronic form) were enough to create the clinical picture presented: fever, lethargy, and inappetence. *Geotrichum* species have rarely been reported as a cause of sepsis or disseminated infection in any species, with severe systemic infections affecting immunosuppressed or patients with severe, chronic co-morbidities in humans. For instance, a report from 2015 highlighted the first case of endophthalmitis in a woman with diabetes mellitus, four months after routine eye surgery [25]. However, as mentioned, it is impossible to say whether all the clinical signs in the current case were due to dermal geotrichosis or there was systemic infection also present since the immune system of the horse may have been adversely affected by the previous corticosteroid therapy. Cessation of corticosteroid therapy, with supplementation of vitamins and antioxidants, as well as change of management may have aided resolution of any systemic underlying disease/ infection in this case. Overall, the horse responded well to the topical antimycotic treatment confirming that dermal geotrichosis was a significant cause of morbidity in this case.

The presence of fungi was initially suspected by cytological examination and was confirmed using routine mycological procedures. *Geotrichum* can be easy to identify using cytology, as it forms true hyphae that segment into rectangular arthroconidia which vary in length (4–10 μm). However, in this case, cytology revealed only the presence of yeast-like organisms not clearly attributable to *Geotrichum* [26]. The successful culture in pure growth, of *Geotrichum candidum* from different skin specimens collected at two different time points confirmed the etiology of this skin disease. The lack of fungus seen on histology is attributed to the lack of crusts submitted for examination, as this fungus is known to be keratinophilic [27] and possibly also to the limited number of biopsy samples taken from not ideal sites. The histopathology changes were disappointing as changes were non-specific in character, but consistent with chronic antigenic stimulation of the epidermis with epidermal hyperplasia and inflammation confined to the superficial dermis. Histological descriptions of geotrichosis are lacking in the literature but the changes seen on histology are consistent with those previously described for superficial fungal infections in horses [1] and for geotrichosis in a dog, although the canine case captured ulceration, folliculitis, and furunculosis which were not seen in the samples from this case [16]. The biopsy was collected only from the neck despite extensive skin disease, which highlights the need for clinicians to collect multiple skin biopsy samples from multiple locations so that skin lesions at different stages of the disease (early, late, ulcerated, non-ulcerated) can be assessed. The authors, therefore, recommend for all cases of multifocal or generalized skin disease, multiple skin biopsy samples, and inclusion of crusts be collected as a routine practice.

A limitation of this case was the lack of adequate records from the initial veterinarian so that no written information about previous treatment regimens such as dosage and drug formulas used were available. The owners orally recalled all the reported information. Dermal geotrichosis was a late diagnosis, so it was impossible to tell whether the mycosis was the primary or an opportunistic infection. The biggest limitation was the lack of identification of the fungus in the histological sample, due to sampling error as described. How, when, where and how many skin biopsy samples to collect remains a challenging decision for clinicians [2]. Despite these limitations, this study confirms the risk of developing generalized fungal infections, such as *Geotrichum* infection, in horses after prolonged treatment with corticosteroids, which is a known risk factor for developing secondary infections in both human and equine veterinary medicine [6]. This case also confirms the efficacy of topical antifungal treatments as a useful therapy to resolve fungal skin infections. Moreover, it highlights the potential clinical usefulness of antioxidant therapy in enhancing the immune response [28] in affected animals and of changing the management as a coadjuvant of treatment. Finally, this case report may be useful to enhance the knowledge of horse owners, trainers, and veterinarians not only on geotrichosis but also on the importance of prompt and correct diagnosis of equine skin disease.

## 5. Conclusions

This study describes a rare case of generalized *Geotrichum SPP* dermatitis. Although it is not possible to confirm that dermal *Geotricum* infection represented a primary or an opportunistic infection, exacerbating factors, in this case, included corticosteroid therapy, initial incorrect diagnosis and poor management. The usefulness of utilizing various laboratory tests when investigating skin lesions in animals are highlighted. Skin lesions can be difficult to diagnose and successfully treat because the clinical signs are often similar even if the etiological cause is different (infectious or non-infectious). When a horse exhibits skin disease, a veterinarian should be promptly called, and appropriate samples should be collected, and differential diagnoses offered. Available diagnostic assays include culture for fungi and bacteria, parasitic investigation, cytology, and histological examinations. In the case of suspected fungi disease, multiple skin biopsy samples and crusts should be collected and submitted to the pathology laboratory. Late intervention, incorrect diagnosis, and inappropriate treatment regimens may lead to poor health and sufferance of patients, which should and can be avoided.

## Figures and Tables

**Table 1:** Hematology profile from a horse affected by dermal geotrichosis for two months, with severe dermatitis, exhibiting fever, inappetence, elevated heart and respiratory rate at the time of blood sampling.

Parameters	Value	Normal Range ^1^
WBC (×10^3^/µL)	13.2	6–12
N (×10^3^/µL)	9.85	2–8.5
L (×10^3^/µL)	2.78	1.16–5.1
M (×10^3^/µL)	0.523	0–0.7
E (×10^3^/µL)	0.015	0–0.2
B (×10^3^/µL)	0.011	0–0.1
RBC (×10^6^/µL)	7.84	5.5–9.5
HGB (g/dL)	12.4	8–14
HCT (%)	33.8	24–44
MCV (fl)	43.2	38–49
MHC (pg)	15.8	15–19
MCHC (g/dL)	36.7	37–40

^1^ (Weiss and Wardrop 2010) [13]. N: neutrophils, L: lymphocytes, M: monocytes, E: eosinophils, B: basophils.

**Table 2:** Biochemical profile from a horse affected by dermal geotrichosis for two months, with severe dermatitis, exhibiting fever, inappetence, elevated heart and respiratory rate the time of blood sampling.

Parameters	Value	Normal Range ^1^
Total Protein (g/dL)	8.69	5.2–7.9
Albumin (g/dL)	2.9	2.6–3.7
Globulins (g/dL)	5.79	2.9–3.9
Bilirubin (mg/dL)	3.03	1–2
AST (U/L)	204	226–366
ALT (U/L)	14	3–23
GGT (U/L)	0	4.3–13.4
SAP (U/L)	370	143–395
CK (U/L)	86	119–287
LDH (U/L)	532	162–412
Urea (mg/dL)	58.1	10–24
Creatinine (mg/dL)	1.7	1.2–1.9
Calcium (mg/dL)	11.44	11.2–13.6
Phosphorus (mg/dL)	3.6	3.1–5.6
Magnesium (mg/dL)	1.78	2.2–2.8
Glucose (mg/dL)	100	75–115
Cholesterol (mg/dL)	72	75–150
Triglycerides (mg/dL)	48	4–44

^1^ (Weiss and Wardrop 2010) [13].

**Table 3:** Serum protein electrophoresis results from a horse affected by a dermal geotrichosis for two months, with severe dermatitis, showing also fever, inappetence, elevated heart and respiratory rate at the time of blood sampling.

Parameters	Value (g/dl)	Value (%)	NR (%) ^1^
Albumin	2.69	30.97	45–60
α1	0.19	2.19	4–6
α2	1.09	12.52	7–13
β1	1.09	12.57	7–9
β2	0.98	11.30	4.5–8.5
γ	2.65	30.45	8–22
Alb/Glo	0.45		0.8–1.0

^1^ (Dietz and Huskamp 1999) [14].

## References

[1] Scott D.W., Miller W.H., Scott D.W., Miller W.H. (2011). Bacterial skin diseases. Equine Dermatology.

[2] Lloyd D.H., Littlewood J.D., Craig J.M., Thomsett L.R. (2003). Practical Equine Dermatology.

[3] Ainsworth G.C., Austwick P.K.C. (1973). Fungal Disease of Domestic Animals. Commonwealth Agricultural Bureau.

[4] De Hoog G.S., Guarro J., Gene J., Figueras M.J. (2000). Atlas of Clinical Fungi. Centraal Bureau Voor Schimmel Cultures.

[5] Cafarchia C., Figueredo L.A., Otranto D. (2013). Fungal diseases of horses. Vet. Microbiol..

[6] Figueredo L.A., Cafarchia C., Otranto D. (2011). Geotrichum candidum as etiological agent of horse dermatomycosis. Vet. Microbiol..

[7] Faravelli G., Conturba B., Mantelli F., Costanti E. (2004). Equine onychomycosis in Northen Italy: A research identifying the aetiological agents. Ippologia.

[8] Kuwano A., Tanaka K., Kawabata M., Ooi Y., Takahashi R., Reilly J.D. (1999). A survey of white line disease in Japanese racehorses. Equine Vet. J..

[9] Keller M., Krehon S., Stanek C., Rosengarten R. (2000). Keratinopathogenic mould fungi and dermatophytes in healthy and diseased hooves of horses. Vet. Rec..

[10] Riond B., Wenger-Riggenbach B., Hofmann-Lehmann R., Lutz H. (2009). Serum protein concentrations from clinically healthy horses determined by agarose gel electrophoresis. Vet. Clin. Pathol..

[11] De Hoog G.S., Guarro J. (1995). Atlas of Clinical Fungi. Centraal Bureau Voor Schimmel Cultures.

[12] D’Hue Z., Perkins S.M., Billings S.D. (2008). GMS is superior to PAS for diagnosis of onychomycosis. J. Cutan. Pathol..

[13] Weiss D.J., Wardrop K.J. (2010). Shalm’s Veterinary Hematology.

[14] Dietz O., Huskamp B. (1999). Handbuch Pferdepraxis.

[15] Sidhu R.K., Singh K.B., Jand S.K., Joshi D.V. (1993). Cutaneous geotrichosis in a dog and its handler—A case report. Indian J. Anim. Health.

[16] Reppas G.P., Snoeck T.D. (1999). Cutaneous geotrichosis in a dog. Aust. Vet. J..

[17] Spanoghe L., Devos A., Viaene N. (1976). Cutaneous geotrichosis in the red flamingo (*Phoenicopterus ruber*). Sabouraudia.

[18] Padalino B., Loy J., Hawson L., Randle H. (2019). Effects of a light-colored cotton rug use on horse thermoregulation and behavior indicators of stress. J. Vet. Behav..

[19] Azarpeykan S., Dittmer K.E., Gee E.K., Marshall J.C., Wallace J., Elder P., Acke E., Thompson K.G. (2016). Influence of blanketing and season on vitamin D and parathyroid hormone, calcium, phosphorus, and magnesium concentrations in horses in New Zealand. Domest. Anim. Endocrin..

[20] Pottier I., Gente S., Vernoux J.P., Gueguen M. (2008). Safety assessment of dairy microorganisms: Geotrichum candidum. Int. J. Food Microbiol..

[21] Miceli M.H., Diaz J.A., Lee S.A. (2011). Emerging opportunistic yeast infections. Lancet Infect. Dis..

[22] Dolensek E.P., Napolitano R.L., Kazimiroff J. (1977). Gastrointestinal geotrichosis in six adult gorillas. J. Am. Vet. Med. Assoc..

[23] Chahota R., Katoch R., Mahajan A., Verma S. (2001). Clinical bovine mastitis caused by *Geotrichum candidum*. Vet. Arhiv..

[24] Henrich T.J., Marty F.M., Milner D.A., Thorner A.R. (2009). Disseminated *Geotrichum candidum* infection in a patient with relapsed acute myelogenous leukemia following allogeneic stem cell transplantation and review of the literature. Transpl. Infect. Dis..

[25] Myint T., Dykhuizen M.J., McDonald C.H., Ribes J.A. (2015). Post-operative fungal endopthalmitis due to Geotrichum candidum. Med. Mycol. Case Rep..

[26] Koneman E.W., Koneman E.W. (2006). Koneman’s Color Atlas and Textbook of Diagnostic Microbiology.

[27] Rajak R.C., Parwekar S., Malviya H., Hasija S.K. (1991). Keratin degradation by fungi isolated from gelatin factory in Jabalpur. Mycopathologia.

[28] Fekete S.G., Kellems R.O. (2007). Interrelationship of feeding with immunity and parasitic infection: A review. Vet. Med..

